# Biomechanical Constraints Underlying Motor Primitives Derived from the Musculoskeletal Anatomy of the Human Arm

**DOI:** 10.1371/journal.pone.0164050

**Published:** 2016-10-13

**Authors:** Valeriya Gritsenko, Russell L. Hardesty, Mathew T. Boots, Sergiy Yakovenko

**Affiliations:** 1 Department of Human Performance, School of Medicine, West Virginia University, Morgantown, West Virginia, 26506, United States of America; 2 Department of Mechanical and Aerospace Engineering, Benjamin M. Statler College of Engineering and Mineral Resources, West Virginia University, Morgantown, West Virginia, 26506, United States of America; 3 Centers for Neuroscience, School of Medicine, West Virginia University, Morgantown, West Virginia, 26506, United States of America; University of Alberta, CANADA

## Abstract

Neural control of movement can only be realized though the interaction between the mechanical properties of the limb and the environment. Thus, a fundamental question is whether anatomy has evolved to simplify neural control by shaping these interactions in a beneficial way. This inductive data-driven study analyzed the patterns of muscle actions across multiple joints using the musculoskeletal model of the human upper limb. This model was used to calculate muscle lengths across the full range of motion of the arm and examined the correlations between these values between all pairs of muscles. Musculoskeletal coupling was quantified using hierarchical clustering analysis. Muscle lengths between multiple pairs of muscles across multiple postures were highly correlated. These correlations broadly formed two proximal and distal groups, where proximal muscles of the arm were correlated with each other and distal muscles of the arm and hand were correlated with each other, but not between groups. Using hierarchical clustering, between 11 and 14 reliable muscle groups were identified. This shows that musculoskeletal anatomy does indeed shape the mechanical interactions by grouping muscles into functional clusters that generally match the functional repertoire of the human arm. Together, these results support the idea that the structure of the musculoskeletal system is tuned to solve movement complexity problem by reducing the dimensionality of available solutions.

## Introduction

Movements are the product of interactions between neural control signals and the musculoskeletal dynamics that depend on limb anatomy [1]. This complex dynamical system depends on the active and passive forces that arise directly or indirectly from muscle contractions and segmental inertia, and requires complex control by the neural motor system. The skeletal limb structure can simplify the control complexity, for example locomotor dynamics is stabilized by advantageous passive dynamics [2]. Musculoskeletal morphology has traditionally been viewed as an additional complexity with redundant characteristics that the central nervous system (CNS) is required to solve [3]. However, evidence has been mounting for the simplifying role of muscle anatomy through increased stability due to viscoelastic properties, which help resist perturbations [4–8]. These properties may even contribute to shaping the multidimensional and state-dependent control parameter space for volitional movements in the “uncontrolled manifold" theory [9]. In particular, Kutch and Valero-Cuevas have suggested that muscular anatomy may help reduce the dimensionality of control space through mechanical coupling even in the absence of a common neural command [10]. However, the extent and topography of muscle coupling across more than several muscles has not been previously described. In the current study, we have used an inductive data-driven approach to further test this idea and to quantify the dimensionality reduction accomplished by the mechanical coupling of muscle actions across the physiological range of arm and hand postures using a validated dynamic musculoskeletal model [11–13].

Muscles have been traditionally classified into agonist and antagonist pairs using their anatomy [14,15] or innervation and participation in sensory-evoked actions [16,17]. For example, stimulation of sensory pathways activates ilia-psoas, tibialis anterior, and extensor digitorum longus that together participate in flexion of hip and ankle of the lower limb [7]. Using this definition, excitation and inhibition patterns give the physiological binary membership of muscles in mutually-opposing functional groups. This idea has been extended further to the concept of motor primitives or synergies, where a smaller subset of grouped muscle actions can accomplish a variety of tasks [18–22]. Alternatively, the anatomical joint-based nomenclature can be used to identify muscle actions around specific joints. For example, the biceps brachii and triceps brachii act as antagonists around the elbow, because the former causes elbow flexion, while the latter causes elbow extension. The latter definition does not rely on neural activations and is purely due to the anatomy of muscle origins and insertions on the bone and their moment arms around the joints. In this study, our goal was to quantify mechanical coupling that underlies the basic functionality and dimensionality of the musculoskeletal system and represents the lowest hierarchical level of movement control. This coupling constrains neural actions and, thus, bears directly on the concept of motor primitives or synergies.

## Methods

### Model

**Fig 1:**
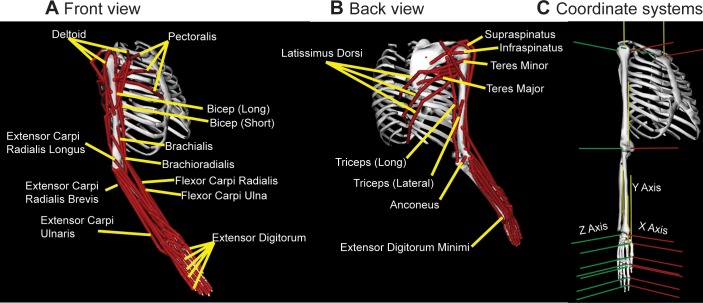
Illustration of the model and local coordinate systems. (A) and (B) Musculotendinous paths from anatomical origins to insertions on the skeleton are illustrated with red lines with selected labels. (C) Coordinate systems for each segment are illustrated with the color-coded cartesian exes in red, yellow, and green for x-, y- and z-axes respectively. Euler angles around these axes represent joint angles. The illustrated posture of the model corresponds to all joint angels at zero. The local coordinate systems are shown only for thumb and index finger. The coordinate systems of the other digits follow the orientation of the coordinate systems for the index finger.

The musculoskeletal model based on the dynamic upper limb model created by Saul et al.(2015)[11] was constructed in OpenSim (version 3.0, Stanford University, Stanford, CA, USA) (Fig 1) and modified in several aspects. Separate bodies for each segment of the hand digits were created to recreate an additional 16 DOFs of the human hand. Metacarpals of digits 2 through 5 (index through little fingers) were modeled as a single body with the inertia of a right rectangular prism. All carpometacarpal joints but the first one were represented by a single wrist joint with 2 DOFs. These corresponded to the rotations between the fused metacarpals 2–5 and ulna coordinate systems around the x-axis for flexion/extension (Fig 1C). Pronation and supination was achieved by the rotation of radius around ulna as in the published model. The first carpometacarpal joint of the thumb was modeled with 2 DOFs. These corresponded to the rotations between the first proximal phalanx and radius coordinate systems around the x-axis for flexion/extension and around the Z axis for abduction/adduction. A single DOF (flexion/extension) was assigned to all metacarpophalangeal joints corresponding to the rotations around the x-axes of the coordinate systems of the proximal phalanges 2–5 and the corresponding metacarpals (Fig 1C). Phalanges were modeled as cylinders with lengths and radii of a human subject. A single DOF (flexion/extension) was assigned to all proximal and distal interphalangeal joints. The axes of rotations of all joints of the arm, with the exception of pronation/supination of the forearm, were adjusted to correspond to Euler angles between adjacent body Cartesian coordinate systems (Fig 1C) to maximize the utility of this analysis for forward and inverse dynamics, where the motion is described in terms of changes in joint Euler angles caused by muscle and inertial torques. The total number of model DOFs, including the arm and hand, was 23. The list of abbreviated names of musculotendinous actuators included in the model and the muscles they represent is in Table 1. Two intrinsic hand muscles, the Opponens Pollicis (OP) and Flexor Pollicis Brevis (FPB), were added to the published model, with their origin and insertion points estimated from Gray’s anatomy [23].

**Table 1:** The abbreviations of muscles included in the analyses.

Muscle Abbreviation	Muscle Name	Muscle Abbreviation	Muscle Name
DELT_A	Deltoid (anterior)	FCR	Flexor Carpi Radialis
DELT_L(AT)	Deltoid (lateral)	FCU	Flexor Carpi Ulnaris
DELT_P	Deltoid (posterior)	PALL	Palmaris Longus
SSPI	Supraspinatus	PTER	Pronator Teres
ISPI	Infraspinatus	PQUAD	Pronator Quadratus
SSCAP	Subscapularis	FDS5	Flexor Digitorum Superficialis (5th digit)
TERMI	Teres Minor	FDS4	Flexor Digitorum Superficialis (4th digit)
TERMA	Terer Major	FDS3	Flexor Digitorum Superficialis (3rd digit)
PECM_R	Pectoralis Major (rostral)	FDS2	Flexor Digitorum Superficialis (2nd digit)
PECM_M	Pectoralis Major (medial)	FDP5	Flexor Digitorum Profundus (5th digit)
PECM_C	Pectoralis Major (caudal)	FDP4	Flexor Digitorum Profundus (4th digit)
LATD_R	Latissimus Dorsi (rostral)	FDP3	Flexor Digitorum Profundus (3rd digit)
LATD_M	Latissimus Dorsi (medial)	FDP2	Flexor Digitorum Profundus (2nd digit)
LATD_C	Latissimus Dorsi (caudal)	ED5	Extensor Digitorum (5th digit)
CORBR	Coracobrachialis	ED4	Extensor Digitorum (4th digit)
TRI_LO	Triceps (long)	ED3	Extensor Digitorum (3rd digit)
TRI_LAT	Triceps (lateral)	ED2	Extensor Digitorum (2nd digit)
TRI_M	Triceps (medial)	ED_M	Extensor Digitorum Minimi
ANC	Anconeus	EIND	Extensor Indicis
SUP	Supinator	EPL	Extensor Pollicis Longus
BIC_LO	Biceps Brachii (long)	EPB	Extensor Pollicis Brevis
BIC_SH	Biceps Brachii (short)	FPL	Flexor Pollicis Longus
BR	Brachialis	APL	Abductor Pollicis Longus
BRR	Brachioradialis	OP	Opponens Pollicis
ECR_LO	Extensor Carpi Radialis Longus	FPB	Flexor Pollicis Brevis
ECR_BR	Extensor Carpi Radialis Brevis	ECU	Extensor Carpi Ulnaris

### Human subjects

This research was approved by the West Virginia University Institutional Review Boards (IRBs) for Protection of Human Research Subjects (protocol number 1311129283A004). Informed written consent was obtained on the forms approved by IRBs from 10 healthy young human subjects. The subjects were 5 males and 5 females of mean age 26.2 ± 6.2 (standard deviation, SD) years, mean weight 77.5 ± 14.1 kg, and mean height 1.74 ± 0.04 m. In addition to participant height and weight, the lengths of all major arm segments represented as individual bodies in our model were measured (Table 1). These measurements were used to scale the model (subject 0) to the dimensions of each individual (subjects 1–10). Each of the model segments and origins and insertions of all muscles were scaled proportionally to the length of each subject’s segment [12].

### Analysis of mechanical coupling

**Fig 2:**
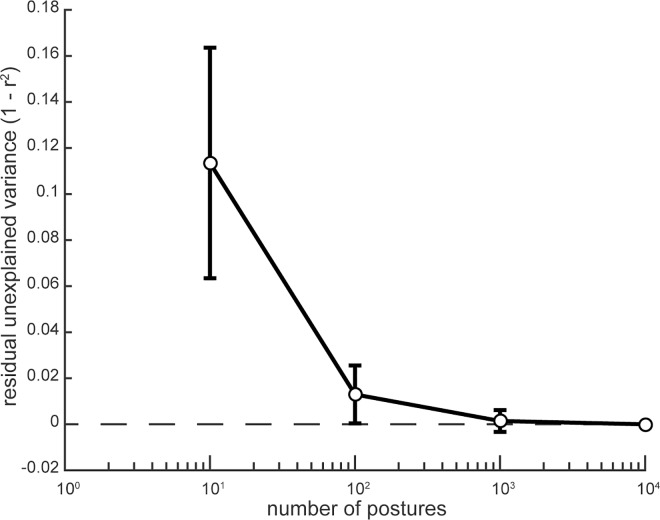
The difference between r^2^ values for the correlations between muscle lengths as a function of the number of selected postures. Error bars show standard deviations around the mean.

We calculated musculotendinous lengths (referred to as muscle lengths) across the full range of motion of the arm using MATLAB (MathWorks Inc.) pipeline tools of OpenSim by permuting postures through all joint excursion combinations within the physiological range of motion in 20% increments. The obtained muscle length data about each of the DOFs at each posture for each muscle of each individually scaled model were then passed through a regression analysis to explore the relationships between muscle lengths for each subject. In this analysis, the correlation coefficients (r) for muscle lengths between all pairs of muscles across all postures were calculated. Due to computational limitations associated with the multidimensional datasets, a random selection of up to 10,000 postures to describe all possible arm and hand state variations was used for the mechanical coupling analysis (see below). Postures when both shoulder abduction and flexion angles were above 90 degrees were excluded from the analysis due to limitations of a gimbal joint. All correlations between muscle lengths were done using 1,000 postures randomly selected from the full dataset. This number of postures was selected because the residual unexplained variance (1—r^2^) at this and higher numbers of postures approached zero (Fig 2).

The agonistic and antagonistic relationships between the muscles of each subject were quantified using hierarchical clustering of the muscle length correlation matrix in MATLAB. Hierarchal clustering was applied to all muscles and separately to only distal muscles. The criterion for inclusion into distal (hand-related) or proximal (shoulder-related) clusters was the level of muscle length correlation between the muscle of interest and either the muscles spanning the shoulder joint or the muscles spanning the wrist joint in all subjects. For clustering, the correlation matrix was transformed into the heterogeneous variance explained (HVE) as described next. The transformation ensured that agonist muscle pairs grouped together, i.e. had small distance values in proportion to shared variance, whereas antagonist muscles appeared relatively far apart, i.e. had larger distance values. Agonist muscles were characterized by positive r-values, and antagonists were characterized by negative r-values. The coefficient of determination (r^2^) was used in the HVE equation as the measure of shared variance between the changes in lengths of muscle pairs. The HVE for agonists was thus set to be equal to (1—r^2^), while the HVE for antagonists was equal to (1 + r^2^). This resulted in agonist muscle pairs with large positive r-values being defined by short distances close to 0, while antagonist muscle pairs with large negative r-values were defined by long distances close to 2. Zero or insignificant correlations were defined by intermediate distances close to 1. Hierarchical clustering was applied using the linkage function with unweighted average distance method to the HVE matrix to identify between 2 and 20 clusters in each subject. The reliability of clustering was evaluated based on the number of muscles that did not fall into the same cluster across subjects. Trivial results with single-muscle clusters were excluded from the reliability analysis.

Unless otherwise stated, all data is referenced by mean ± SD.

## Results

**Fig 3:**
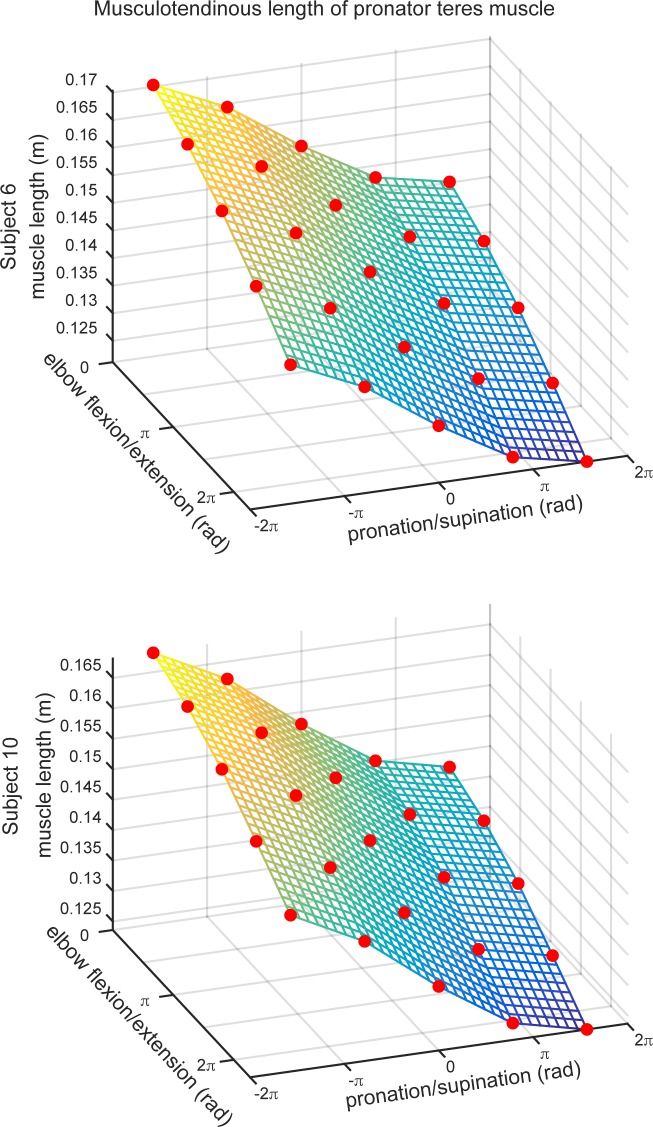
Examples of muscle lengths for the pronator teres, a single 2-DOF muscle originating on the humerus and attaching on the radius, in two subjects. The data points (circles) correspond to muscle lengths throughout the physiological range of motion for each DOF.

The musculoskeletal model comprised 52 musculotendinous actuators (model muscles) that spanned 23 DOFs. Of the 52 actuators, 26 represented compartments of 7 muscles, e.g. 3 triceps actuators representing long, lateral, and medial heads of the triceps brachii. Thus, the model represented the anatomical arrangement of 33 individual muscles. There were 15 actuators that spanned only the shoulder joint (3 DOFs), 3 actuators that spanned both the shoulder and elbow (4 DOFs) joints, 6 actuators that spanned only the elbow joint (2 DOFs due to flexion-extension and pronation-supination), and 8 actuators that spanned both the elbow and wrist (3 DOFs, not including pronation/supination) joints, with the remaining 20 actuators spanning the wrist and at least 1 finger joint. Thus, most muscles were associated with several DOFs. For example, the length of the pronator teres depends on the angles of forearm pronation/supination and elbow flexion/extension shown in Fig 3. The lengths of the actuators changed non-linearly as a function of the DOFs they controlled, as do their moment arms [24]. This implies that a constant activation of a given muscle results in a different contribution of that muscle to the net joint torque when the arm is held at different postures or throughout the motion. These non-linearities are the result of complex anatomical paths that the muscles take as they wrap around each joint, particularly joints with multiple DOFs.

The action of each musculotendinous actuator in the model depends on its attachment to the bones and the path it takes around the joint. These data are based on human anatomical data [11]. To investigate the effect of individual skeletal proportions on mechanical coupling, the lengths of arm segment were scaled to the values from each of 10 human subjects. This changed the values for muscle lengths associated with each arm posture. The skeletal proportions across subjects varied with SD, ranging from 5% to 27% of the average segment length (Table 2). However, the relationships between muscle lengths were highly stable across subjects, as described in detail in the following sections.

**Table 2:** The summary of anthropometric measurements. All distance measurements, unless indicated otherwise in brackets, were made between the estimated centers of joint rotation.

Segment name	Length (m)	Length (% of subject height)
Thorax	0.217 ± 0.032	12.5 ± 1.9
Shoulder (between clavicle and scapula acromial tip)	0.194 ± 0.016	11.2 ± 0.8
Humerus	0.279 ± 0.026	16.1 ± 1.4
Ulna	0.262 ± 0.014	15.1 ± 0.8
Radius	0.262 ± 0.014	15.1 ± 0.8
Hand (mean metacarpal length of phalanges 2–5)	0.085 ± 0.009	4.9 ± 0.5
First metacarpal	0.046 ± 0.009	2.7 ± 0.5
First proximal phalanx	0.0369 ± 0.004	2.1 ± 0.3
First distal phalanx	0.0276 ± 0.004	1.6 ± 0.2
Second proximal phalanx	0.046 ± 0.005	2.7 ± 0.2
Second middle phalanx	0.028 ± 0.003	1.6 ± 0.1
Second distal phalanx	0.023 ± 0.002	1.3 ± 0.1
Third proximal phalanx	0.048 ± 0.009	2.8 ± 0.5
Third middle phalanx	0.033 ± 0.004	1.9 ± 0.2
Third distal phalanx	0.024 ± 0.002	1.4 ± 0.1
Forth proximal phalanx	0.043 ± 0.010	2.5 ± 0.0
Forth middle phalanx	0.031 ± 0.005	1.8 ± 0.2
Forth distal phalanx	0.023 ± 0.002	1.3 ± 0.0
Fifth proximal phalanx	0.035 ± 0.008	2 ± 0.4
Fifth middle phalanx	0.023 ± 0.006	1.3 ± 0.3
Fifth distal phalanx	0.012 ± 0.003	1.1 ± 0.2

**Fig 4:**
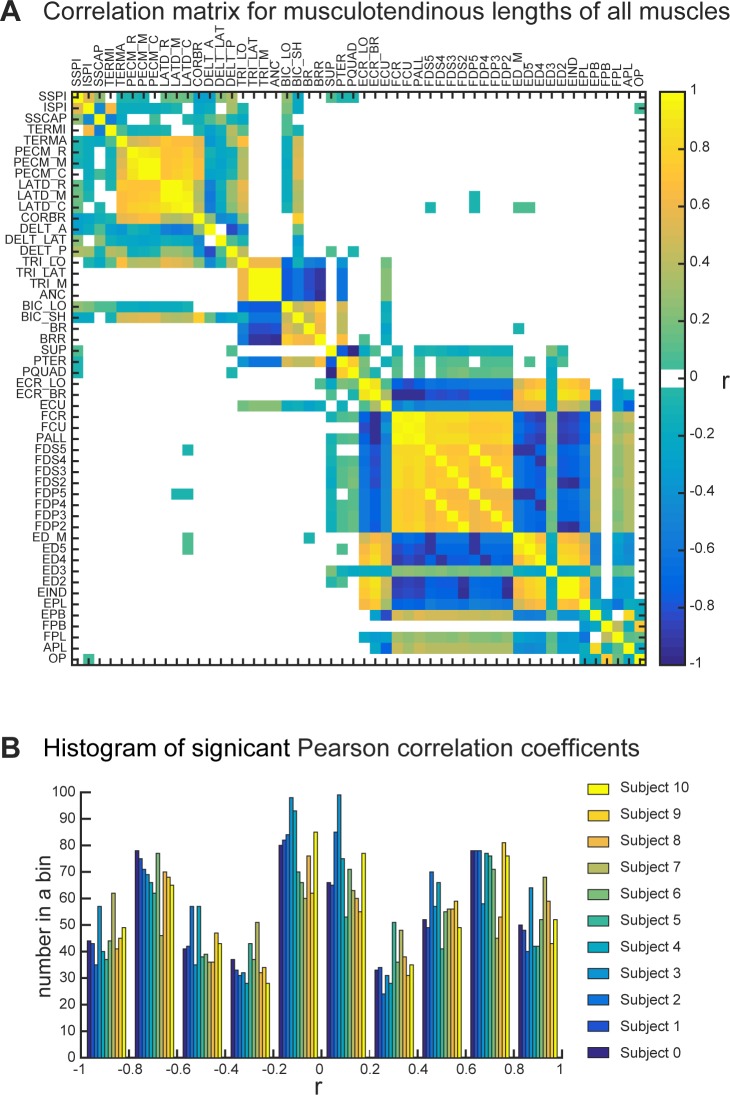
Examples of the correlations between muscle lengths in a single subject. Only significant correlations are plotted (p < 0.05). (A) Pearson correlation coefficient (r) between muscle lengths of all muscle pairs. Blue colors indicate negative correlations; yellow colors indicate positive correlations. (B) Histogram of *r*-values for each subject across all muscle pairs. The bar plots are binned with 0.2 increments, and only significant values were included in the analysis.

As expected, the muscle lengths across muscles were highly correlated in agonistic or antagonistic fashion (Fig 4A). Positive correlations indicate that the muscle length increases or decreases together, representing agonistic action across multiple arm postures (Fig 4B). Here, the method is limited to the examination under the isometric condition that does not take into account dynamics or history-dependent muscle properties [25–27]. Negative correlations indicate coincident increase of one muscle length while the other is decreased, representing antagonistic action. Not surprisingly, the lengths of all actuators representing compartments of the same muscle were highly correlated (bright yellow squares around the unity line in Fig 4A). Surprisingly, however, most of the muscles showed strong correlations that broadly formed two large clusters, where proximal muscles of the arm were correlated with each other and distal muscles of the arm and hand were correlated with each other, but not as much with the proximal cluster. For example, the length of LATD_M was highly correlated with that of PECM_C (r^2^ = 0.594), but the correlation with the distal cluster was minimal (r^2^ = 0.004 with FDP5). Similarly, the length of ED5 was highly correlated with that of ED_M (r^2^ = 0.793), but the correlation with the proximal cluster was minimal (r^2^ = 0.004 with LATD_C). This is the first time the agonistic and antagonistic actions of muscles have been quantified across the whole workspace of the human arm.

**Fig 5:**
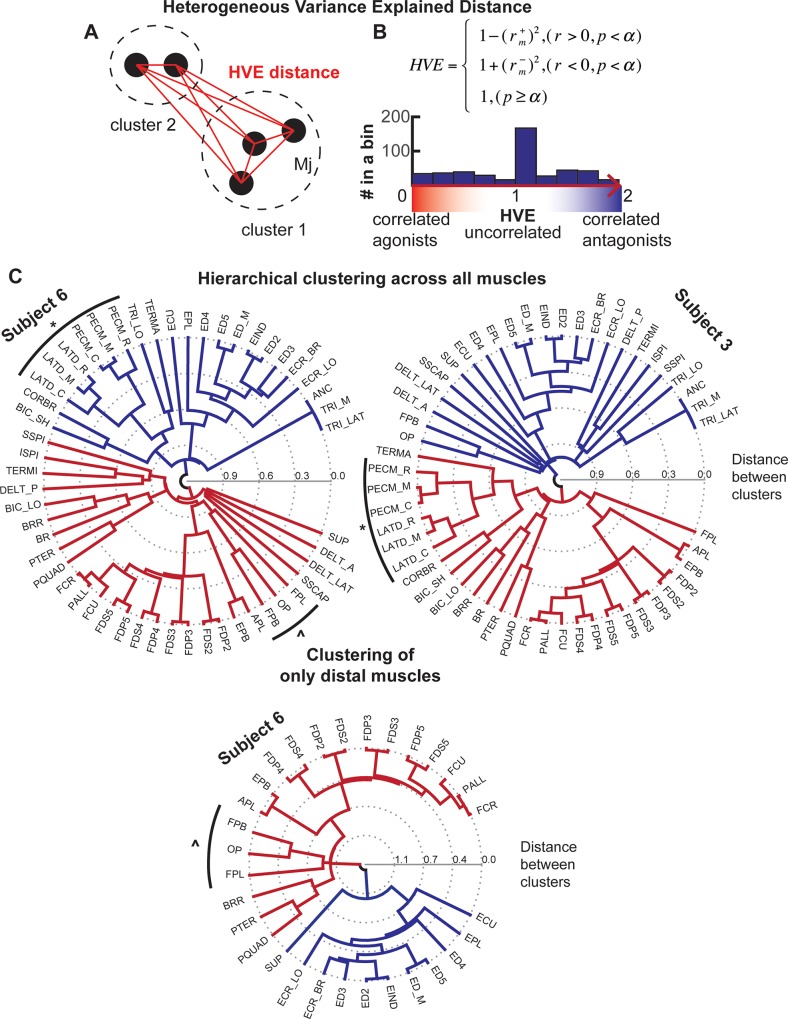
Hierarchical clustering methodology and examples for two subjects. (A) Geometric illustration of heterogenous variance explained (HVE). HVE distance is determined by the correlations of musculotendon length between muscle pairs determined by the equation in (B). (B) The equation for calculating HVE distance. The negative regressions (r^-^) indicate opposite or antagonistic actions of muscle pairs, when the positive ones (r^+^) correspond to the synergistic or agonistic actions. Insert shows a histogram of HVE values for one subject across all muscle pairs. (C) Examples of hierarchical clustering for individual subjects. Clustering across all muscles is shown in the top two polar dendrograms. The bottom plot shows clustering across only the distal muscles for one of the subjects. Lines emanating from the center indicate the distance between muscle clusters calculated from HVE. The main agonist-antagonist division can be established using a high clustering threshold (2 clusters with dark red and dark blue lines), and further subdivisions are revealed by the progressive lowering of the threshold. Example matching clusters are marked by outside brackets with * or ^.

The hierarchical clustering analysis of muscle lengths quantitatively identified muscle groups at multiple levels of detail. The first 2 clusters in all subjects represented broadly flexor and extensor actions across all joints or DOFs (Fig 5C, dark blue and red clusters emanating from the center). However, two groups were insufficient for the consistent classification of all muscles across subjects. Some muscles may be classified differently for different subsets of subjects. For example, the subgroup that contains latissimus dorsi and pectoralis major was clustered either with extensors in 5 out of 11 subjects or with flexors in the rest of the subjects (see Fig 5C, subgroup marked * in two different subjects). Note that the composition of this subgroup remained unchanged. The separate analysis of distal musculature showed the same pattern of clusters as the analysis of all muscles. For example, the same subgroup consisting of thumb muscles remained unchanged in both analyses (see Fig 5C, subgroup marked **^** in the same subject).

**Fig 6:**
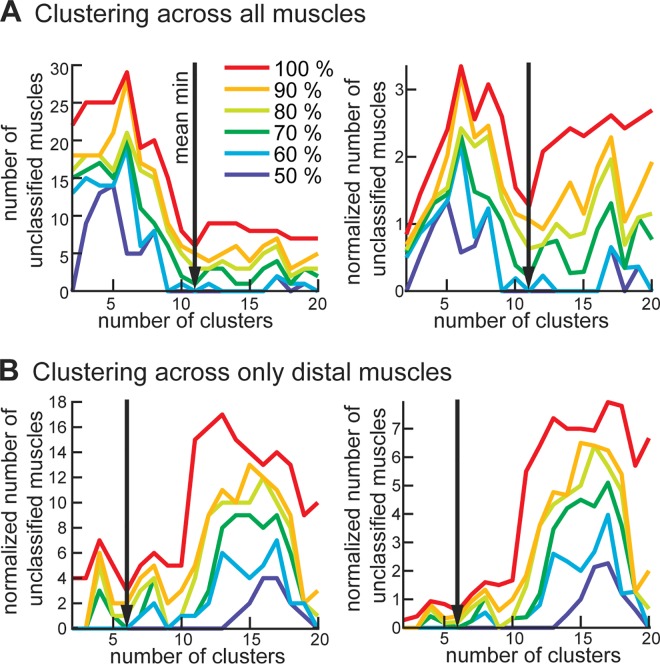
Reliability of clustering across subjects. (A) The average number of unclassified muscles is shown as a function of the number of clusters. Each colored line corresponds to the level of stringency for the variability in classification across subjects, e.g. 100% stringency corresponds to the same classification in all subjects. The right panel shows the same values normalized to the average number of muscles in all clusters. (B) The same analysis as in A for distal muscles only. Vertical black arrow indicates the nontrivial minimum for the number of clusters (11 clusters for all and 6 clusters for distal muscles), which represents the most reliable number of muscle clusters.

**Fig 7:**
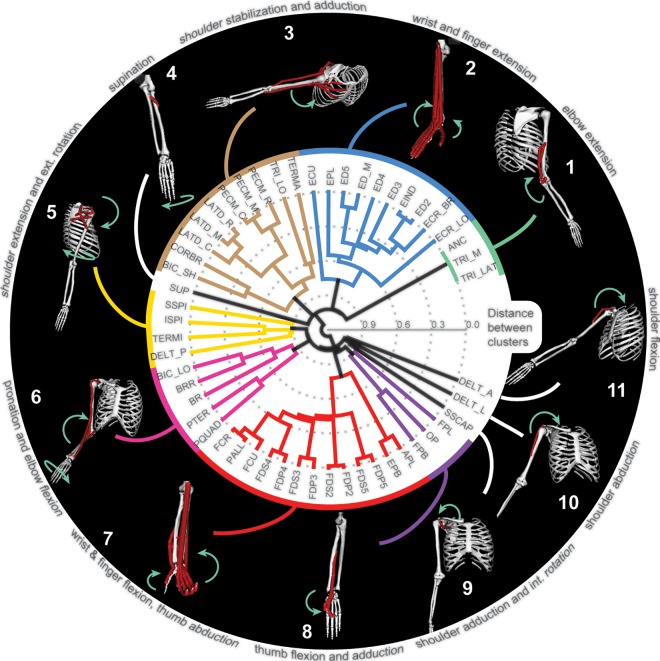
Mean hierarchical clustering across all subjects. The polar dendrogram illustrates hierarchical clustering as described in Fig 5C. Inserts along the perimeter illustrate the directions of motion (green arrows) produced by the activation of muscles in the model shown in Fig 1. Only muscles that belong to the corresponding cluster are shown on each insert.

The consistency of muscle cluster assignment across subjects changes as a function of the number of clusters selected in the analysis (Fig 6). The number of unclassified muscles was generally high when muscles were divided into 3 to 8 clusters, which means less consistent clusters across subjects (Fig 6A). This followed by a plateau of 9 to 13 more consistent clusters, in which the same muscle groups were identified across subjects. Further subdivision into more than 13 clusters generated increasingly more trivial results with single-muscle clusters, which is evidenced by increasing normalized number of unclassified muscles (Fig 6A, right plot). When the inclusion threshold for cluster assignment across subjects was increased from 50% (muscle belongs to the same cluster in 50% of subjects) to 100% (muscle belongs to the same cluster in all subjects), the number of unclassified muscles changed for the different numbers of clusters. All muscles were classified into the same clusters in at least half of all subjects when 2 or 9–16 clusters were selected (Fig 6A, dark blue line). The increase in the inclusion threshold to 100%, i.e. the muscle had to belong to the same cluster across all subjects, increased the peak number of unclassified muscles from 15 to 30 (Fig 6A red line on left plot). The most reliable number of clusters, based on the minimal number of unclassified muscles across all thresholds, was 11 (Fig 6A, black arrows). Normalizing the number of unclassified muscles to cluster size did not change this estimate (Fig 6A, right). Similar trends were seen in the reliability of clustering of distal muscles (Fig 6B). Here, the most reliable number of clusters was 6 (Fig 6B, black arrows). This analysis identified the minimum number of reliable clusters, which are illustrated on the mean polar dendrogram across all subjects in Fig 7. These clusters of muscles that span multiple joints represent the simplest actions that can be accomplished through mechanical coupling.

## Discussion

In this work, we have described for the first time the low-dimensional structure of agonistic or antagonistic mechanical actions, termed *the mechanical coupling*, of major arm and hand muscles across their physiological range of motion. We demonstrated that a low-dimensional structure emerges even from the musculoskeletal anatomy without the presence of common neural feedforward or feedback signals (Fig 7). We found that there exists an optimal range for the number of clusters that reliably group muscles according to actions (Fig 6). Thus, these results may help us address the unresolved controversies associated with the definition of motor primitives by detailing the lowest level in the bottom-up organization of the motor control system. This mechanical coupling between muscles defines the natural repertoire of actions that the musculoskeletal system can produce in presence of inertial and gravitational forces, external perturbations, and neural control signals. Therefore, our results provide further evidence to support the idea that musculoskeletal anatomy helps to reduce the dimensionality of control space through the mechanical coupling [4–8,10].

One prevalent theoretical explanation of how the nervous system resolves limb control problems is based on the idea of *motor primitives*, i.e. groups of muscles sharing the same common source of neural activation [19–22]. Inherent in this concept is the idea that motor primitives reduce the complexity of neural control signals by enabling the production of any movement from a smaller selection of control actions [19,20]. However, the theory of motor primitives, or synergies, defined this way has recently come under increased scrutiny due to the indivisible interaction and mutual dependency between neural control of muscle activations and biomechanics of the resulting movement [28]. These interactions and dependencies may emerge in the synergy analyses when limb movement engages sensory feedback from mechanically coupled muscle groups [8] or, alternatively, constitute evidence for common feedforward drive within neural code [29–31]. The common neural drive would also originate if the neural networks are embedding movement dynamics for processing motor commands. The concept of central pattern generators (CPG) in the spinal cord, in particular, is a representative example of low-dimensional neural processing for rhythm generation that is coupled to mechanical oscillations between limbs and the environment to produce locomotion [6]. Also, the evolving predominant view is that neural processing can be represented by a dynamical system acting through available neuromuscular elements to generate appropriate signals for desired movements [32]. Taken together, neural activity within the hierarchical CNS contains the representation of downstream processing that may reflect the low-dimensional representations of targeted mechanisms resulting in neural signals consistent with the idea of common drive.

The neuromechanical tuning may be used to redefine motor primitives in terms of individual actions being controlled. The hierarchal structure of both the neural motor system and the mechanical coupling implies that the control complexity can be broken down into specific actions produced by common signals to muscle groups at different levels of the identified mechanical coupling hierarchy. Then, CPGs in the spinal cord, which are modeled as a dynamical system [1], could be viewed as neural motor primitives that are entrained with the inverted pendulum oscillator formed by the mechanical interactions of limbs with the ground [6,33,34]. Because the entraining originates in the sensors associated with muscles, the musculoskeletal organization has bearing on this unit of control. The CPG generates antagonistic activity that results in gross mechanical oscillatory actions through interactions between antagonistic groups of muscles [35]. The CPGs are also thought to contribute to arm motor control [6,33,34,36,37]. The antagonistic groups observed in our analysis as the first two clusters in the mechanical coupling diagram may reflect the same concept (Fig 5C). When dexterous movements are required, e.g. to step over obstacles during locomotion or reaching, the gross CPG motor primitive must be fractioned into smaller components specific to the task [30]. In our analysis, this would be equivalent to following the polar dendrogram from the center with *gross* representations to periphery with fractured *fine* representations (Fig 7). The neighboring fine motor primitives in our analysis could be combined to represent functional movements. Defensive limb movements can be generated by three combinations of 10, 5, and 6 groups; feeding movement can arise from the recruitment of all groups in 6–8; and the manipulation movements can be generated by four combinations 2, 6, and 11, followed by 7 for grasping. While these combinations are qualitatively similar to those observed in response to the long-train intracortical microstimulation of the motor cortex [38–40], the link between neural activity and the composition of coupled muscle groups remains to be tested in future studies.

Another result in this study is the salient separation between muscle motor primitives of proximal and distal arm joints. This is unexpected, because the subsets of proximal and distal muscles span the same elbow joint and contribute to pronation/supination DOF. Only sparse correlations between the pairs of muscles spanning primarily proximal and primarily distal joints are present in our study (Fig 4). This result indicates that the anatomical arrangement of muscles is consistent with the idea of two distinct control targets: proximal arm and distal hand groups. Coincidentally, the spatiotemporal separation between the activation of proximal and distal muscles is present in goal-directed reaching movements that are traditionally separated into two phases: gross arm motion to transport the hand to the desired location and fine hand motion to manipulate objects. It has also been suggested that these phases are controlled separately by the nervous system [41,42]. Such muscle organization and the possible separation within neural control pathways may be the result of evolutionarily-driven expansion of distal musculature to enable the increased dexterity of object manipulation characteristic of primates. The spatiotemporal separation of muscle activity during limb transfer, generally controlled by proximal muscles, and limb placement, generated by distal musculature, is also evident in the regulation of evolutionarily connected phases of reaching movement and precise modifications in quadruped stepping. Moreover, these separate temporal phases are correlated to the activity of distinct corticospinal circuits [30].

Our analysis uses the incidence of length excursions in different postures as a measure of functional similarity in muscle actions. The analysis is based on sampling representative postures within the physiological range of motion (ROM); yet, this posture space may not be functionally homogenous. It included both likely and unlikely joint configurations based on the frequency of observing their representation in daily use [43,44]. For these subsets of joint configurations there may exist distinct relationships within subsets of muscles. The method of uniform sampling used here may not capture the coupling or uncoupling among the muscle pairs within these subsets of likely and unlikely postures. Then there may also be a subset of muscles with changing relationships within different postures. Because these muscle pairs would have low correlations in our analysis, the only groups that could be affected would be those associated with the weak relationships between antagonistic muscles acting on scapular (groups 3 & 5 of Fig 7). Fig 4 shows that these are the only large groups with r-values within medium to low correlations, i.e. between -0.5 and 0.5 values, that may be affected. It is tempting to speculate that the proximal arm muscles may change their functional affiliation based on the familiarity with task. This could be reflected in different biomechanical advantages or affordances that influence movement planning [45]. This question will be addressed in the future research. In the presented analysis, the correlations across postures indicate the shared dependence on joint constraints to define functionally similar muscles over the full physiological ROM that includes all possible limb postures with the exclusion of extremes.

Several methods are commonly used to derive motor primitives from muscle activity, and all rely on extracting shared signal redundancy among neural discharge and/or muscle activity [21,30,46–49]. Cumulatively, these studies support the idea that muscle motor primitives are reflected in the neural activity; however, the confounding factors may offer alternative explanations for coupled activity [8,10,50]. The mechanical coupling derived from the correlations of muscle lengths across physiological postures qualitatively matches the groups observed in the decomposition analyses. For example, the biceps long, brachioradialis, brachialis, and pronator teres are in the same muscle group 6 (Fig 7) and are also part of the W1 synergy identified with time-varying synergy analysis [51]. Similarly, the teres major and latissimus dorsi are part of a single muscle group 3 and posterior deltoid is a part of an adjacent group 5 identified through the mechanical coupling analysis (Fig 7) and are also part of the W5 synergy identified with time-varying synergy analysis [51]. This result is consistent with observations that the underlying musculoskeletal dynamics can constrain the space of neural commands to a low-dimensional subspace identified with decomposition methods [10]. Thus, the existence of the mechanical coupling of muscles generally agrees with the findings of alternative methods.

In conclusion, our analysis of arm and hand muscles is a quantitative description of the functional organization within the musculoskeletal system that contributes to the concept of motor primitives. The organization of movement derived from the musculoskeletal architecture offers a novel perspective on the motor control problem solved by CNS.
