# Correction: Biomechanical Constraints Underlying Motor Primitives Derived from the Musculoskeletal Anatomy of the Human Arm

**DOI:** 10.1371/journal.pone.0203968

**Published:** 2018-09-07

**Authors:** Valeriya Gritsenko, Russell L. Hardesty, Matthew T. Boots, Sergiy Yakovenko

The third author's name is spelled incorrectly. The correct name is: Matthew T. Boots.
